# Endodontic Treatment of a Taurodontism Tooth: Report of a Case

**Published:** 2006-10-01

**Authors:** Shahrzad Nazari, Farshid MirMotalebi

**Affiliations:** 1 *Department of Endodontic, Dental School, Hamedan University of Medical Sciences, Hamedan, Iran*; 2 *Department of Prosthodontic, Dental School, Hamedan University of Medical Sciences, Hamedan, Iran*

**Keywords:** Endodontic Treatment, First Molar, Taurodontism

## Abstract

Taurodontism is a rare dental anomaly in which the involved tooth has an enlarged and elongated body and pulp chamber with apical displacement of the pulpal floor. Endodontic treatment of these teeth is challenging, because it is hard to identify the number of root canals. In this article a case of bilateral involvement of maxillary first molars is presented. Endodontic treatment of right maxillary first molar with taurodontism was indicated due to irreversible pulpitis. This article describes the procedures of root canal therapy.

## INTRODUCTION

Taurodontism (Bull-like tooth) is a rare morphologic variation which causes the occluso-apical elongation of the chamber, and the reduction of the root size. This variation is thought to be caused by the failure of Hertwig’s epithelial sheath diaphragm in invagination at the proper horizontal level, resulting in a tooth with short roots, an elongated body, an enlarged pulp chamber, and a normal dentin (1).

Preliminary literatures have related this anomaly to an unidentified type of ectodermal malformation. It can also be related to amelogenesis imperfecta or even genetic abnormalities. Today, it is considered as an anatomic variance that could occur in the normal population (2).

Taurodontism can express itself as a single entity or as a part of a syndrome. So such syndrome can be diagnosed first by a dentist because of the presence of a taurodont tooth.

The clinical size of a taurodont tooth is usually normal while molars are involved with greater propensity in permanent dentition. Almost half of the cases show this condition bilaterally, and has a high prevalence in Middle Eastern and skimoo tribes (3). Radiographic evaluation can reveal this condition because of its unique appearance with an occluso-apicaly enlarged pulp chamber, short roots and absence of cervical constriction (4).

Taurodontism, although not common, is an important occurrence that may influence dental management of patients. The present case describes endodontic treatment of a maxillary first molar with taurodontism.

## CASE REPORT

**Figure 1:**
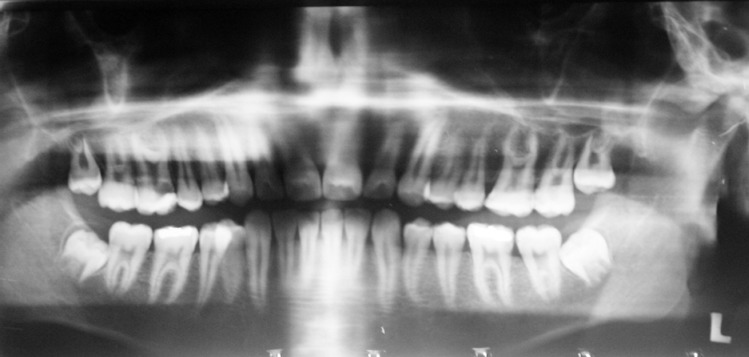
Orthopantumographic image of the patient

The patient was a 17 years old boy with severe pain on the right maxillary first molar who was referred with orthopantomograph radiography (Figure 1). An urgent treatment (pulpotomy) had been performed on the tooth.

**Figure 2:**
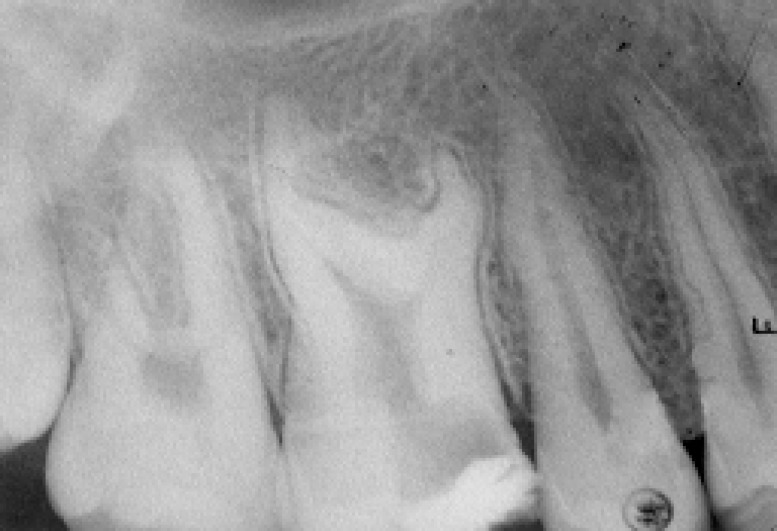
Periapical radiographic images of right maxillary first molar

A periapical radiograph of the affected tooth showed a pulp chamber and three short roots with the furcation area migrated towards the apex; indicating hypertaurodontism (Figure 2) (Shifmann and Chanannel classification) (5).

His medical and familial history revealed no systemic disorders.

The patient had a history of orthodontic treatment because of the crowding of his teeth in both jaws. According to patient records, the etiology of the crowding had been referred to tooth size, arch size discrepancy (Bolton discrepancy).

**Figure 3:**
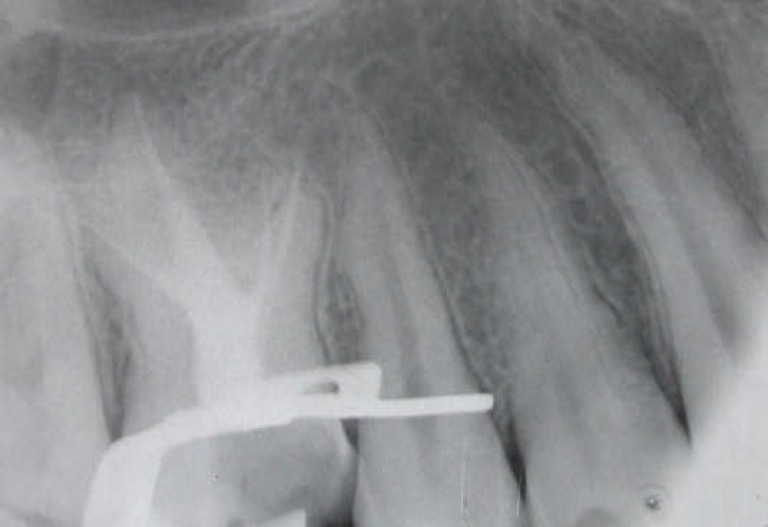
Periapical radiographic images immediately after root canals obturation

Hypertaurodontism on the upper left side was confirmed by an orthopantomograph. In this radiograph, both mandibular first molars had normal root configuration (Figure 1).

**Figure 4:**
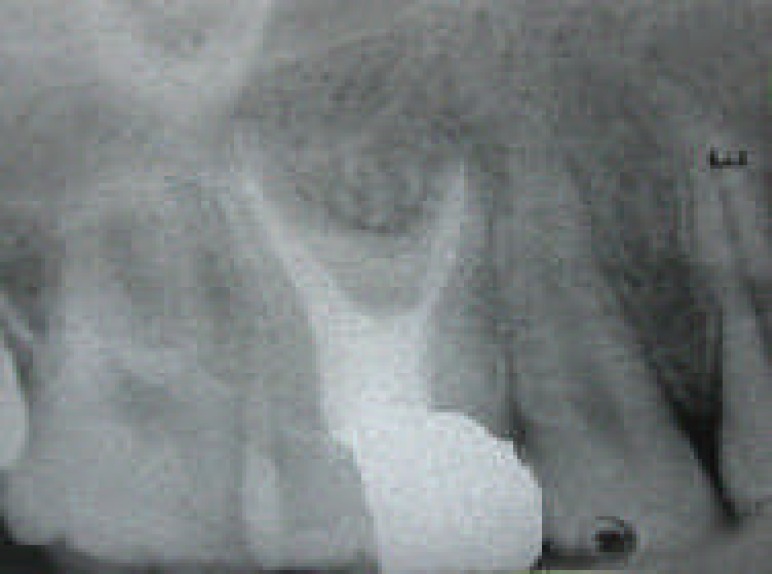
Radiographic image of the tooth one year after a complete root canal therapy

After the injection of local anesthesia the temporary filling material was removed. The access cavity was extended to the mesial and distal walls of the pulp chamber. The tooth was isolated by rubber dam, and with a little effort three initial files were set in the root canals and then the initial radiograph was taken. During the instrumentation a wide palatal canal (P), and two narrow mesiobuccal (MB) and distobuccal (DB) canals were found. An electronic apex locator (Raypex 4, VDW GmbH, Munich, Germany) was used to determine the initial working lengths. At this point, a second periapical radiograph was taken. In the radiograph examining an extra root in the buccal side of the mesiobuccal root was found. The working length determination radiograph, which was taken with a file in the suspected root canal, verified its presence. Instrumentation was carried out using a rotary system (M two, VDW GmbH, Munich, Germany).The palatal and buccal root canals were instrumented up to #50 and #30 files respectively. Copious irrigation with 2.5% sodium hypochlorite was used as irrigating solution.

A modified obturation technique was used because of the complexity of the inner root canal anatomy and the proximity of the buccal orifices. This consisted of combined cold lateral compaction in the apical part with warm vertical compaction in the elongated pulp chamber with an obturator unit (Endo-Twin2, VDW, GmbH, Munich, Germany).

The final radiograph confirmed a well compacted root filling material extending to the predetermined length in each of four root canals (Figure 3). The restorative dentist was recommended to avoid post placement and use other techniques for tooth reconstruction. The recall appointments were adjusted at 6 and 12 months later. At 6 months, the patient was asymptomatic and at one year follow-up visit, the radiographic examination showed healthy periodontium and periradicular tissue (Figure 4).

## DISCUSSION

Taurodontism is frequently associated with other anomalies and syndromes (4). In this case, the patient was healthy and without any known diseases.

Endodontic treatment in taurodontism teeth has been described as a complex and difficult procedure.

Cohen and Taintor presented 5 cases of taurodontism. Of these, 2 cases required root canal therapy which was difficult. The number of root canals varied in cases (6). Hayashi reported a case of taurodontism which had five root canals.

Although all orifices were found, but only two root canals were instrumented and filled to the apex (7).

Tsesis et al. reported a successful endodontic treatment of a left maxillary first molar with four root canals (8).

## CONCLUSION

The present case describes successful completion of endodontic treatment of a taurodont maxillary first molar, which seemed impossible to perform with conventional techniques.
